# Mechanistic Insight Into the Roles of Integrins in Osteoarthritis

**DOI:** 10.3389/fcell.2021.693484

**Published:** 2021-06-18

**Authors:** Hongfu Jin, Shigang Jiang, Ruomei Wang, Yi Zhang, Jiangtao Dong, Yusheng Li

**Affiliations:** ^1^Department of Orthopedics, Xiangya Hospital, Central South University, Changsha, China; ^2^National Clinical Research Center for Geriatric Disorders, Xiangya Hospital, Central South University, Changsha, China; ^3^Department of Orthopedic Surgery, The Third Hospital of Hebei Medical University, Shijiazhuang, China; ^4^Department of Endocrinology and Metabolic Diseases, The Fourth Affiliated Hospital of Anhui Medical University, Hefei, China

**Keywords:** integrin, osteoarthritis, cartilage, subchondral bone, mechanical signal conduction, synovial membrane

## Abstract

Osteoarthritis (OA), one of the most common degenerative diseases, is characterized by progressive degeneration of the articular cartilage and subchondral bone, as well as the synovium. Integrins, comprising a family of heterodimeric transmembrane proteins containing α subunit and β subunit, play essential roles in various physiological functions of cells, such as cell attachment, movement, growth, differentiation, and mechanical signal conduction. Previous studies have shown that integrin dysfunction is involved in OA pathogenesis. This review article focuses on the roles of integrins in OA, especially in OA cartilage, subchondral bone and the synovium. A clear understanding of these roles may influence the future development of treatments for OA.

## Introduction

As the most common degenerative joint disease, OA can destroy both cartilage and subchondral bones, causing progressive degeneration of the articular cartilage and subchondral bone, as well as the synovium (Chen et al., 2017). The unique composition and structure of the cartilage extracellular matrix (ECM) allows for the long-term load-bearing capabilities of the joint, playing important roles in joint function. OA can affect the ECM, causing increased catabolic activity and inflammation changes in the mechanical function of the ECM in the joint (Guilak et al., 2018). There is evidence that metabolic changes in the ECM play an vital role in the pathological process of OA (Rahmati et al., 2017). As participants in an integral membrane complex, integrins play important roles in the transmembrane association, which are involved in the ECM and the cytoskeleton interactions and take part in transmembrane signals conduction. Previous studies have shown that integrin dysfunction is involved in OA pathogenesis. This review article focuses on the roles of integrins in OA, especially in OA cartilage, and subchondral bone, as well as the synovium. A clear understanding of these roles may influence the future development of treatments for OA.

## What Are Integrins?

Integrins, comprising a heterodimeric transmembrane protein family, contain two subunits (the α subunit and β subunit). There are eighteen α subunits and eight β subunits. All these can combine into twenty-four integrin molecules (Ansari and Byrareddy, 2016). The integrin molecules can act as transmembrane receptors to bind ECM proteins, which can regulate essential physiological functions of cells, such as adhesion, migration, the inflammatory response, and mechanical signal conduction. Depending on the types of ligands, integrins can be divided into two categories: Arg-Gly-Asp (RGD)-binding receptors and non-RGD-binding receptors. Non-RGD binding receptors include collagen-binding receptors, laminin-binding receptors, and leukocyte-binding receptors (Margadant and Sonnenberg, 2010; Finney et al., 2017). As transmembrane molecules, integrins play important roles in the physiological function of cells. Integrins can mediate adhesion between cells and their surroundings. Integrin-based adhesion is formed mainly between the cytoskeleton and the ECM. For example, lamellipodia and filopodia, the bumps of the cell surface and cytoskeleton, can attach to the ECM through integrin-based adhesions (Geoghegan et al., 2019). By combining with intracellular proteins, like α-actinin, vinculin, and paxillin, integrins can connect the inner cytoskeleton to the ECM (Zaidel-Bar et al., 2004). Cell signaling mediated by integrins can regulate the functions of cells, including their matrix remodeling, adhesion, migration, and mechanical signal conduction (Loeser, 2002). In addition, integrins, working in concert with the cytoskeleton, can receive external mechanical stimulation and transmit information on the mechanical status of the ECM into the cell. As mechanical sensors, integrins play important roles in facilitating cell movement, generating tension on the ECM, activating intracellular signaling pathways, and producing biological reactions (Humphries et al., 2019; Sun et al., 2019).

More and more results showed that the dysregulated function of integrins was implicated in OA pathogenesis. Animal experiments showed that α4, α5, and α2 integrin expression was increased in cartilage and that the content of proteoglycan and fibronectin was also changed (Almonte-Becerril et al., 2014). High levels of α1β1 and α3β1 were detected in OA cartilage tissues, potentially facilitating the modulation of ECM deformation and promoting chondrocyte hypertrophy (Häusler et al., 2002). The components of the ECM play important roles in maintaining chondrocyte homeostasis. For example, the stiffness of collagen in cartilage is associated with the occurrence and development of OA, not only on the joint surface but also at the interface between cartilage and bone (Wen et al., 2012). Collagen type II (COLII) can suppress chondrocyte hypertrophy and deterioration of OA by promoting the interaction between β1 integrin and drosophila mothers against decapentaplegic protein 1 (SMAD1) (Lian et al., 2019). Because of the crosstalk between the cartilage and subchondral bone, the subchondral bone of OA patients is also changed. Compared to controls, subchondral osteocytes showed a series of changes in cell morphology, such as rough cell surfaces, unorganized dendrites, and so on (Jaiprakash et al., 2012). Studies have also shown that culturing bone cells on the ECM of OA specimens leads to reduced expression of integrin β1 and inactivation of the FAK cell signaling pathway (Prasadam et al., 2013). The changes of αVβ3 integrin level can vary with the degree of cartilage degeneration in patients with OA (Wang et al., 2018). Dysfunction of integrin αvβ3 and integrin-associated protein (CD47) signaling pathways have been proved that can promote the occurrence and progression of OA (Wang et al., 2019). We will discuss the role of integrins in OA in detail in the following text.

## Integrins in Articular Cartilage and Chondrocyte Homeostasis

### Integrins in Chondrocytes Adhesion

Articular cartilage, composed mainly of water, collagen, proteoglycans, and cells, provides a smooth surface for joints and facilitates the transmission of loads (Ulrich-Vinther et al., 2003; Carballo et al., 2017). The articular cartilage lining the surface of the subchondral bone is multi-layered. The surface layer consists of collagen fibrils and chondrocytes, which parallels to the articular surface. In the deeper layer, the arrangement of collagen fibrils is more random and collagen fibrils are vertically inserted into the subchondral bone in the deepest layer (Silver et al., 2001). Chondrocytes, constituting the main cell group of adult articular cartilage cells, play important roles in maintaining the balance between the anabolism and catabolism of the ECM (Loeser, 2009; Kozhemyakina et al., 2015; Li et al., 2017; Liang et al., 2018; Kadry and Calderwood, 2020). Under normal physiological conditions, the ECM components are in a slow renewal state, which maintains homeostasis between chondrocyte catabolism and anabolism. Studies have confirmed that integrins, such as α1β1, α2β1, αVβ3, αVβ5, and so on, are expressed on chondrocytes (Loeser et al., 2000; Kurtis et al., 2003; Lahiji et al., 2004; Shattil et al., 2010). The interactions between chondrocytes and the ECM mediated by integrins are crucial for chondrocyte activity. ECM, as an “informative” environment, is made up of many molecules, including COLII, proteoglycans (PGs), hyaluronic acid (HA), and chondroitin sulfate (CS), etc., And the various components in the ECM are important for the structure and function of the ECM (Gao et al., 2014; Hansen, 2019). ECM changes in OA seem to be driven by the imbalance between anabolic and catabolic activities of chondrocytes, which are responsible for the occurrence and development of OA. The increase of catabolism in ECM was observed in OA pathology (Rahmati et al., 2017).

As a transmembrane molecule of chondrocytes, integrin plays an important role in cartilage homeostasis. Integrins act as a central regulator in multicellular biology, which can coordinate with multiple cellular functions. The integrins can mediate cell adhesion between chondrocytes and the ECM (Ginsberg, 2014; Dustin, 2019; Kadry and Calderwood, 2020). Integrins and their connections to the cytoskeleton play important roles in monitoring cell adhesion and the physical properties of the ECM (Romero et al., 2020). Cell adhesion can be achieved by binding the adhesion superstructures with integrins to the periphery of the non-collagenous fibril (Woltersdorf et al., 2017). Chondrocytes express several integrin protein families, like fibronectin (α5β1), COLII and COLVI (α1β1, α2β1, α10β1), laminin (α6β1), osteopontin (αVβ3), and so on (Loeser, 2000). Chondrocytes can be attached to various cartilage and bone proteins, which is mainly mediated by integrins, including members of the β1 and β3 subunit family. The regulation of chondrocyte adhesion is related to the activation or increase of integrin expression (Loeser, 1993). Adhesions between cartilage oligomeric matrix protein (COMP) and chondrocytes occurs through αVβ3 integrin (Chen et al., 2005). α10β1 integrin, expressed by normal adult chondrocytes, can bind COLII, and α1β1 integrin can also bind COLII collagen but preferentially binds COLVI collagen (Camper et al., 1998; Loeser et al., 2000). Complex interactions between integrins and their extracellular ligands show that integrins play important roles in chondrocyte adhesion.

### Integrins in Chondrocyte Mechanotransduction

Studies have shown that the mechanical stress environment of joints is an essential factor affecting or regulating chondrocyte activity *in vivo* (Loeser et al., 2000). Mechanical load plays an important role in the formation, differentiation, shaping, maturation and matrix synthesis of cartilage. Chondrocytes are exceedingly sensitive to mechanical changes in their surroundings. The stabilizing maintenance of articular cartilage can be regulated by stimulations, such as mechanical load, small soluble molecules in ECM and matrix components. Mechanical stimulation can be divided into dynamic compression, fluid shear, tissue shear, and hydrostatic stimulation (Sharifi and Gharravi, 2019). Integrins, as an important mechanical receptor, can affect the physiological function of chondrocytes by activating the mechanical signal pathway, a process known as mechanotransduction (Roca-Cusachs et al., 2012; Geoghegan et al., 2019).

The integrin-mediated biochemical signals of extracellular mechanical stimuli are dependent on integrin-matrix interactions (Zhao Z. et al., 2020). Studies have shown that integrin α1β1 is a crucial molecule for transducing mechanical load (Jablonski et al., 2014). The periodic mechanical load can significantly facilitate the fibronectin-integrin α5β1 bond (Kong et al., 2013). Periodic mechanical load activates downstream protein kinase C (PKC) signals by stimulating chondrocytes α5β1 integrin, which can cause hyperpolarization of chondrocyte membrane (Wright et al., 1997). Mechanical signal pathways mediated by integrins are involved in the proliferation and matrix synthesis of chondrocytes, such as integrin β1-Src- GIT ArfGAP 1 (GIT1)- focal adhesion kinase (FAK) (Tyr576/577)- extracellular regulated protein kinase 1/2 (ERK1/2), integrin β1-FAK(Tyr397)-ERK1/2, and integrin β1- Ca2+/calmodulin dependent protein kinase II (CaMKII)- Proline-rich tyrosine kinase 2 (Pyk2)-ERK1/2 signal pathway (Liang et al., 2017; Ren et al., 2018). Studies suggested that the death signaling pathway mediated by integrins also participated in the process that excessive mechanical load acting on cartilage explants (Jang et al., 2014).

### Integrins in Chondrocyte Transmembrane Signaling

In addition to being involved in mechanical signal transduction, integrin involvement in transmitting signals has attracted attention (Loeser, 2014; Prein and Beier, 2019). The cytoplasmic signaling within chondrocytes, called “inside-out signaling,” can regulate the affinity of integrins for their ligands. The combination of the α subunit and β subunit cytoplasmic tails can maintain integrins in an inactive state. Signals from G-protein-coupled receptors can activate integrins, causing phosphorylation of the cytoplasmic domain of the β subunit, which can disrupt the combination of the α subunit and β subunit (Takada et al., 2007). Through “inside-out signaling,” the adhesion intensity and strength between integrins and the ECM can be regulated. Binding to specialized extracellular ligands, integrins can be activated by “outside-in signaling.” In this situation, integrins cluster on the surface of the cell and undergo conformational changes that activate cytoplasmic kinase and cytoskeletal signaling cascades. The cross-talking of signaling mechanism components in integrin-mediated “outside in” and “inside out” signaling pathways play a role in maintaining cartilage homeostasis (Attur et al., 2000). As a vital mediator of between chondrocytes and ECM in cartilage, integrins can regulate the response to signals emitted from the ECM, which play an important role in cell proliferation, survival, differentiation and matrix remodeling.

**FIGURE 1:**
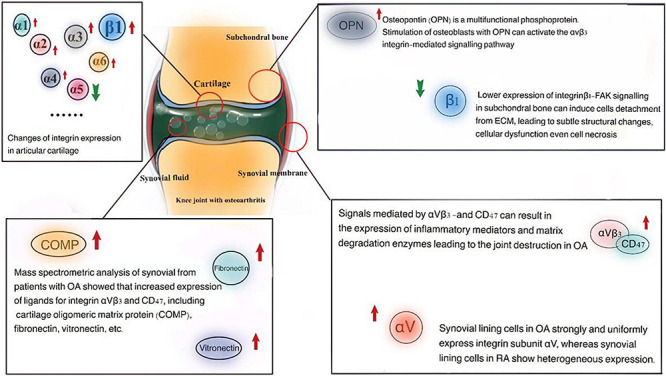
Integrins undergo many changes in OA, suggesting integrins participate in pathological processes of OA. Recent studies have identified the important role of integrins in OA cartilage, as well as subchondral bone and synovium.

Studies have shown that integrin-mediated signaling pathways are involved in the gene expression of micro-molecules, like inflammatory mediators, chemokines, matrix metalloproteinases (MMPs), such as MMP-1, MMP-3, MMP-10, MMP-13, etc., (Werb et al., 1989). The α5β1 integrin, an important cellular membrane receptor of chondrocytes, can be activated by proteins with RGD peptide, antibodies against α5β1 integrin or fibronectin fragments (Fn-fs) in ECM. One reason for the imbalance between anabolism and catabolism of chondrocytes is that the combination of α5β1 integrin with soluble Fn-fs. Fn-fs, generated by MMPs degrading fibronectin (Fn), have catabolic properties. The pro-catabolic response to matrix fragments may be particularly associated with the destruction of ECM. RGD-containing Fn-fs, when binds to α5β1 integrin, was found to be the most active (Homandberg et al., 1993). PKCδ is the rate-limiting factor at the convergent points of signaling input from Fn-fs. PKCδ activation can cause the activation of nuclear factor kappa B (NF-κB) in addition to MAP kinase (MAPK) (Lee et al., 2013). MAPK activation can lead to inhibition of anabolic signaling, suppression of PG production, and upregulation of catabolic proteases, like MMP-3, MMP-13, and so on. Many signaling pathways are interconnected, which can enhance cartilage destruction in OA. For example, MAP3-kinase TGF-β-activated kinase 1 (TAK1) can link MAPK signals to the activation of NF-κB, which may play a role in OA pathogenesis (Cheng et al., 2016). The NF-κB pathway, considered a typical proinflammatory signaling pathway, plays an important role in many inflammatory diseases (Lawrence, 2009). Both pathways work together to inhibit anabolic signaling and stimulate ECM degeneration (Figure 1). All these can stimulate chondrocytes to produce proinflammatory mediators, such as prostaglandin E2 (PGE2), reactive oxygen species (ROS), a disintegrin and metalloproteinase with thrombospondin motif (ADAMTS)-5, nitric oxide (NO), and MMPs (Arner and Tortorella, 1995; Homandberg, 1999; Forsyth et al., 2002; Gemba et al., 2002). The roles of integrins in pathological processes of OA will be discussed in detail in the following text.

## Changes in Integrin Expression and Functional Behavior in Osteoarthritis

Cartilage surface defects are common changes in OA. Chondrocytes can be fixed to special positions by adhesion, which in turn can trigger the secretion of molecules that repair the defect and tissue. Eventually, chondrocytes adhere to the host tissue and become part of the cartilage. There are many important molecules involved in chondrocyte adhesion to the ECM, such as Annexins (mainly A5), CD44, and integrins. Studies found that there was an increased level of α1β1, α3β1, α2β1, α4β1, and α6β1 in cartilage tissue of OA (Loeser et al., 1995; Lapadula et al., 1997; Ostergaard et al., 1998). These changes in integrins may be the result of feedback regulation from changes in the ECM. Growth factors and cytokines can stimulate integrin expression, which accounts for the change in integrins in OA (Loeser, 1997). Dysfunction of integrin αVβ3 and CD47 signaling in chondrocytes has been confirmed to contribute to inflammation and joint destruction in OA (Wang et al., 2019).

**FIGURE 2:**
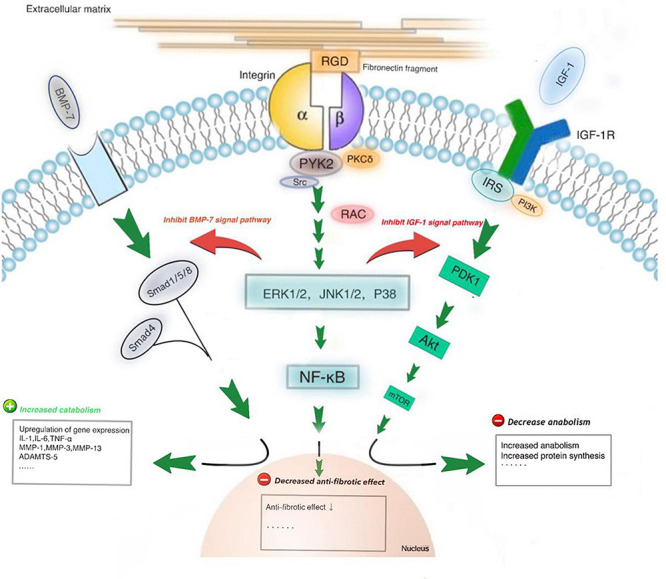
The RGD-containing fibronectin fragments (Fn-fs) can induce cartilage damage and proteoglycan loss. PKCδ is the rate-limiting factor at the convergent point of signaling input from Fn-fs. PKCδ activation can lead to nuclear factor kappaB (NF-κB) activation in addition to MAP kinase (MAPK) activation. MAPKs (ERK1/2, JNK1/2, and p38) activation can lead to inhibition of anabolic signaling, including IGF-1 and BMP7 signaling pathways, increased levels of inflammatory cytokines and upregulation of catabolic proteases like MMP-3 and MMP-13.

Integrin α5 is inferred to be a protective factor that inhibits hypertrophy, OA occurrence, and chondrocyte development. Evidence has shown that the expression of integrin α5 in chondrocytes was lower in an OA model of rats induced by surgery than in a normal group, suggesting that changes of ECM may lead to the imbalance of cartilage homeostasis and affects the repair ability of chondrocytes, finally deteriorating the pathological changes of OA (Castaño Betancourt et al., 2012; Bernhard et al., 2017). Lack of α1 integrin subunit was associated with early degradation of cartilage homeostasis and accelerated aging-dependent lesions. Compared with wild-type (WT) mice, more severe degradation, glycosaminoglycan depletion, and higher expression of MMP-2 and MMP-3 in the cartilage of α1-KO mice (Zemmyo et al., 2003). In addition, the increase of α2 and α3 subunits expression in cartilage tissue is related to the degree of fibrosis and a high expression of αV integrin was detected in hypertrophic chondrocytes of rats with OA. All these changes suggest that integrins play important roles in OA (Figure 2).

## The Roles of Integrins in Osteoarthritis

### Integrins in Osteoarthritis Cartilage

#### Changed ECM Components and Integrins

Changed ECM components in OA are a result of an imbalance of synthesis and catabolism, which can serve as initiating or progressive factors of OA (Guilak et al., 2018). Developmental and mature chondrocytes are constantly interacting with ECM and remodeling ECM. Various ECM components promote OA by stimulating receptors on chondrocytes membranes, such as endothelin-1 (ET-1), which induces chondrocyte senescence and cartilage damage by endothelin receptor B, so as integrins (Au et al., 2020). Integrin-mediated signaling pathways are key sources of the catabolic reactions critical for joint destruction in OA. Developmental chondrocytes can express a special molecule called integrin-β-like 1 (Itgbl1) at specific stages, which can inhibit integrin-mediated signal pathways and promote cartilage generation. However, the expression of Itgbl1 was decreased significantly in the chondrocytes of OA (Song et al., 2018). A rat model experiment suggested that Indian hedgehog (Ihh) expression during the late stages of OA can inhibit the endochondral ossification induced by bone morphogenetic protein 7 (BMP-7) and αV integrin (Garciadiego-Cázares et al., 2015). During the procession of OA, ECM-degrading enzymes, such as urokinase-type plasminogen activator (uPA), ADAMTSs, and MMPs, can degrade components of the ECM (Pérez-García et al., 2019). Angiopoietin-like protein 2 (ANGPTL2) secreted by chondrocytes can induce the production of inflammatory factors through the integrin α5β1/MAPKs, Akt, and NF-κB signaling pathways (Takano et al., 2019). Another study found that the stimulation of the αVβ3 and αVβ5 integrins of chondrocytes can upregulate the gene expression of Interleukin-1β (IL-1β), tumor necrosis factor-α (TNF-α), MMP-3, and MMP-13 (Hirose et al., 2020). Animal experiments have shown that ofloxacin can interfere with the β1 integrin/ERK/MAPK signal pathway and thus induces apoptosis in young rabbit articular chondrocytes (Sheng et al., 2008). CD147, also called ECM metalloproteinase inducer (EMMPRIN), is a highly glycosylated transmembrane glycoprotein, which can interact with β1 integrin (α3β1 and α6β1) in the membrane of chondrocytes (Orazizadeh and Salter, 2008). Previous studies suggested that collagen type X (COLX) can interact with chondrocytes directly through major integrin α2β1 (Leitinger and Kwan, 2006).

#### Excessive Mechanical Load and Integrins

The mechanical load can affect the cartilage matrix. Chondrocytes are constantly subjected to external mechanical load, thereby regulating remodeling. The optimal level of mechanical load is essential to maintain the dynamic balance of chondrocyte homeostasis (Vazquez et al., 2019). The mechanical load acting on joints can directly affect the production of matrix degradation enzymes and further affect cartilage homeostasis (Aigner et al., 2006; Goldring and Goldring, 2007). The moderate mechanical load can lead to hypertrophy. The excessive mechanical load can lead to collagen network damage, resulting in irreversible cartilage destruction (Jørgensen et al., 2017). Moreover, excessive mechanical load of cartilage also can cause cartilage tissue damage through necrosis (Arokoski et al., 2000). MAPKs, as central regulators of cell signaling pathways, play important roles in cell physiological functions, which are considered potential targets for the treatment of OA (Loeser et al., 2008). MAPKs can mediate cell signaling pathways induced by the mechanical stimulation of integrins and then regulate chondrocyte gene expression and proliferation in response to the mechanical load acting on joints (Roca-Cusachs et al., 2012). After the stimulation of integrins by mechanical load, a signaling cascade is activated (Lee et al., 2000).

The homeostasis of articular cartilage depends partly on the mechanical load generated in daily activities. Appropriate joint load stimulates chondrocytes to maintain healthy cartilage by producing specific protein components. Conversely, excessive mechanical load alters cartilage composition and causes focal degeneration of cartilage, leading to disease (Smith et al., 2004; Monfort et al., 2006). Excessive mechanical load acting as signals from the ECM can activate integrins, which further promotes the progressive destruction of the cartilage matrix in OA (Fang et al., 2021). Under excessive mechanical load, integrins can regulate the responses of chondrocytes to mechanical stimulation through multiple pathways. Studies have shown that integrins can interact with the MAPK-ERK pathway. Articular chondrocytes respond to α5β1 integrin, acting as a mechanoreceptor. Animal experiments showed that mechanical load led to an increase in the number of α5 subunit in both immature cartilage and mature cartilage, but the number of β1 subunit was not increased (Lucchinetti et al., 2004). Integrin-associated protein (CD47/IAP) can interact with α5β1 integrin to modulate chondrocyte responses to mechanical signals (Orazizadeh et al., 2008). The downstream signaling cascades and cell responses are different in OA chondrocytes. Excessive mechanical signals can regulate key molecules in MAPK signal cascades to maintain their efficacy in proinflammatory environments. For example, mechanical signals can affect gene expression and chondrocyte proliferation during proinflammatory environments through integrin-linked kinase and signal pathways (Perera et al., 2010). All these factors can progressively destroy the cartilage matrix in OA. Cellular communication network factor 2 (CCN2), a cysteine-rich secreted matricellular protein, is highly expressed and secreted into the ECM under mechanical load, regulating cell physiological functions. Integrins, the first receptor to perceive mechanical load on the cell membrane of chondrocytes, can enhance the gene expression of CCN2. CCN2 expression is increased when exposed to excessive mechanical stress, that further triggers cartilage fibrosis through the activation of integrin-mediated signal pathways (Huang et al., 2021). There were significant differences in signal events and cell responses when mechanical load acts on normal and OA chondrocytes (Millward-Sadler and Salter, 2004). Under excessive mechanical stress, integrins can respond to inflammatory activation in chondrocytes. High levels of α1β1 and α3β1 were observed in the cartilage tissues of OA patients, which may potentially contribute to ECM deformation and chondrocyte hypertrophy (Zhao Y. et al., 2020).

#### Cytokine Signals and Integrins

As the most common disease in the elderly, OA can damage the ECM of cartilage, leading to pain and dysfunction of joints. There are many factors that can cause OA, including mechanical injury, cytokines, superoxide release, adipokines, etc., (Sofat, 2009; Zhang et al., 2018). The role of cytokines in OA has gradually drawn people’s attention (Sofat, 2009). Integrins as key receptors on the cell surface can interact with cytokines secreted into the ECM, that may participate in the pathogenesis of OA. The gene expression of integrins can be regulated by cytokines like insulin-like growth factors-1(IGF-1) and transforming growth factor-beta (TGF-β) (Loeser, 2000). Integrins can change their expression patterns under pathological conditions and promote the deterioration of OA by releasing active TGF-β and regulating various signals downstream of the integrins (Zhang et al., 2020). A high level of TGF-β can disrupt cartilage homeostasis and impair the metabolic activity of chondrocytes. Animal studies have shown that knockdown of αV integrin gene in mouse chondrocytes can reverse TGF-β activation and subsequent abnormalities in articular cartilage metabolism (Zhen et al., 2021). Cytokines in ECM are considered to have a variety of effects on cartilage. We listed some of the cytokines associated with integrins in this section. The chemokine CX3CL1 can induce chemotaxis of monocytes, neutrophils, and fibroblasts. CX3CL1 acts through its receptor CX3CR1. By stimulating CX3CR1, CX3CL1 can activate integrin-dependent migration of chondrocytes, which is evident in many articular cartilage diseases (Poniatowski et al., 2017). Angiopoietin-like 2 (ANGPTL2) secreted by chondrocytes can stimulate the integrin α5β1/MAPKs, Akt, and NF-κB signaling pathways leading to ECM degradation and inflammatory response, which plays a negative role in the pathogenesis of OA (Shan et al., 2019). In addition, both growth differentiation factor 5 (GDF-5) and BMP-7 in chondrocyte could regulate the expression of integrins, that may participate in normal physiological function and OA progression (Garciadiego-Cázares et al., 2015).

### Integrins in Healthy and Osteoarthritic Subchondral Bone

Like the bones in other parts of our bodies, subchondral bone osteocytes are the main mechanical sensitive cells in bone. Increasing evidence showed that integrin-based adhesion could promote mechanical transduction and play an important role in forming subchondral bone (Geoghegan et al., 2019). During the formation of subchondral bone, Osteoblasts and osteocytes express β1 subunit, that can combine with α1, α2, α3, α4, and α5 subunits. β3 subunit connects with αv subunit in osteoblasts and osteocytes (Horton et al., 1991; Engleman et al., 1997; Geoghegan et al., 2019). All these integrin molecules are involved in cell-matrix adhesion and facilitate mechanical conduction. Integrin-mediated signaling pathways and their cross-talking with Wnt/β-catenin signaling pathways are involved in osteoblast mechanical transduction (Marie et al., 2014). Mechanical load acting on joints can regulate the metabolism of healthy subchondral bone osteoclasts and cause gene expression of interleukin-6 (IL-6), interleukin-8 (IL-8), MMP-3, MMP-9, MMP-13, etc., (Sanchez et al., 2012). The structure of subchondral bone can determine the mode of mechanical load acting on cartilage and the mode of TGF-β activation, which can regulate the metabolism of chondrocyte and cartilage homeostasis. Mechanical stress can trigger TGF-β activation through αV integrin-mediated signaling pathways. A high level of TGF-β activation has been detected in areas with high mechanical load in cartilage (Zhen et al., 2021).

In addition to degenerative changes in articular cartilage, OA also causes the destruction of subchondral bone. The role of subchondral bone in OA has been gradually recognized (Goldring and Goldring, 2010). The causes of the subchondral bone of OA, specially in non-load-bearing areas, include synovial fluid inflow, mechanical contusion, vascular lesion, etc., (Chan et al., 2017). Abnormal subchondral bone remodeling plays an important role in the pathological changes of OA. Osteocyte morphology was found to be altered in the subchondral bone of OA patients, the cell body became round and roughened by the degeneration of typical dendrites and the appearance of unorganized dendrites (Jaiprakash et al., 2012). OA can cause the destruction of subchondral bone, osteoblast dysfunction of subchondral bone at the cell level; and cystic lesions, sclerosis, and osteophytes at the tissue level (Weber et al., 2019). Risk factors for OA include aging, obesity, abnormal joint mechanical load, and joint sprain, which interact in a complex way (Palazzo et al., 2016). In particular, the excessive mechanical load of joints triggered a series of cell changes, including cartilage damage and subchondral bone adaptation changes (Adebayo et al., 2017). The imbalance between cartilage and subchondral bone destroys the normal physiological relationship between both tissues and further leads to the deterioration of OA. This section of this article focuses on integrins in the subchondral bone of OA.

Pathological changes of subchondral bone were found in OA, including microstructural damage, bone marrow edema-like injury, and bone-cyst formation (Li et al., 2013). Excessive mechanical load applied upon articulation may be critical for these changes. The sclerosis of the subchondral bone is widely regarded as one of the features of OA. Osteoblasts isolated from sclerotic areas of subchondral bone were found to express levels of α5, αv, β1, and β3 integrins and CD44, which is similar to the levels in non-sclerotic osteoblasts under basal conditions (Sanchez et al., 2012). Subchondral bone is hypo-mineralized due to abnormal bone remodeling. Osteopontin (OPN), a multifunctional phosphoprotein, was found that highly expressed in OA tissues. Stimulation of osteoblasts with OPN can activate the αvβ3 integrin-mediated signaling pathway (Su et al., 2015). Culture of osteocytes on defective ECM tissue produced by OA subchondral bone osteoblasts caused a decreased gene expression of integrin β1 and deactivation of the FAK signaling pathway. Many proteins containing the three amino acid sequence RGD in the ECM can be recognized by corresponding integrin β1 receptors (Schaffner and Dard, 2003; Marini et al., 2017). The combination of integrins with these macromolecules can activate a series of downstream signals and initiate a cascade of phosphorylation events, which are essential for the function of subchondral bone cells, such as cell adhesion and proper cytoskeletal organization (Legate et al., 2009; Michael and Parsons, 2020). Lower expression of integrin β1-FAK signaling in the subchondral bone can induce cell detachment from ECM, leading to subtle structural changes, cellular dysfunction even cell necrosis (Prasadam et al., 2013).

### Integrins in Osteoarthritic Synovium

The synovium can secret synovial fluid to joint space, which contributes to the functional properties of articular surfaces and modulation of the state of chondrocytes. For example, hyaluronic acid (HA) secreted by synovial lining cells contribute to the integrity of the cartilage surface and reduce friction at cartilage surface (Hui et al., 2012). Synovitis in OA is characterized by increased angiogenesis and hypoxia (Liu et al., 2019). Fibroblasts and macrophages in the synovial lining are important sources of inflammatory mediators, such as IL-1, IL-6, TNF, etc., The destruction of cartilage can induce the inflammation of the synovium, causing the production of cytokines. The concentrations of cartilage-protecting factors in the synovial membrane decrease, and harmful factors are constantly generated (Scanzello and Goldring, 2012; Hügle and Geurts, 2017; Michael and Parsons, 2020). All these alterations can deteriorate OA by the degradation of the ECM and apoptosis of chondrocytes.

In synovium tissue, the gene expression of integrins depends on the specific cell location and cell type. Most gene expressions of integrins are similar in synovium tissue but differ in the synovial lining, where the fibroblasts and macrophages degrade ECM and invade the cartilage. α6β1 integrin is expressed only by fibroblasts, while macrophages not. The expression levels of α5, αν, and β1 integrin in the synovium lining increased compared to the sub-lining areas (Pirilä et al., 2001; Lowin et al., 2009; Lowin and Straub, 2011). Synovial cells are involved in the protection and maintenance of the stability of joints. Studies on rabbit synovial fibroblasts showed that cooperative signaling mediated by α5β1 and α4β1 integrins plays a dominant role in regulating MMP expression signaling in response to FN. MMP expression can remove the damaged matrix, which is the first step in repairing the damaged matrix. The cross-talking of integrins makes it possible for synovial fibroblasts to identify whether the matrix is intact or damaged. The volume of synovial fluids is increased in the OA articular cavity. Synovial fluids obtained from OA tissue showed increased expression of ligands for integrin αvβ3 and CD47, including COMP, fibronectin, and vitronectin. Increased ligand binding affinity of αvβ3 and CD47 was found in the synovium of the OA rat model. Signals mediated by α_V_β_3_ and CD47 can result in the expression of inflammatory mediators and matrix degradation enzymes, leading to joint destruction in OA (Wang et al., 2019). Integrin αvβ3 and α5β1 are involved in synovial cell proliferation, differentiation, and migration. Both are overexpressed in damaged synovial cells, acting as inflammatory and angiogenic factors in the progression of rheumatoid arthritis (RA). Their roles in the OA synovial membrane need further study (Morshed et al., 2019). There is evidence that the synovial lining cells in OA strongly and uniformly express integrin subunit αv, whereas synovial lining cells in RA show heterogeneous expression. Both RA and OA cells fail to express the integrin subunit β3. These results show different manifestations of the αV and β3 integrin subunits in cytokine-stimulated fibroblast-like cells from the synovium of OA and RA *in vitro* (Rinaldi et al., 1997). All these results showed that integrins are not only play a significant role in synovial joint development, but also involved in the pathological changes of OA.

## Prospects for Integrin Research in the Treatment of Osteoarthritis

Osteoarthritis, with a high incidence in the elderly population, brings tremendous economic burdens to individuals and society. Pain and joint dysfunction are the main causes of decreased quality of life in patients with OA. Current clinical trials mainly include repairing defects of cartilage and bone, intra-articular injections of drugs, physical exercise, etc. However, all the therapies has been proven that don’t significantly have improvement in disease progression and successfully prevent arthroplasty surgery (Grässel and Muschter, 2020). People are constantly looking for new ways of treating OA. Integrins, as important receptors on the cell surface, play important roles in OA, which may provide new targets for the therapies of OA. In this section, we discuss the application prospects for integrin research in the field of OA treatment.

Interfering with the integrin-mediated signaling pathway provides a novel therapeutic approach for OA. For example, osteopontin (OPN) can interact with the integrin αVβ3 receptor, which participates in maintaining the homeostasis of articular cartilage. High expression of OPN was detected in cartilage and synovial fluid, which may be involved in the progression of OA. Recently, researchers have attempted to use this protein as a diagnostic marker of OA or a targeted drug against OA (Cheng C. et al., 2014). Low-intensity pulsed ultrasound (LIPUS) can interfere with integrin - FAK-phosphatidylinositide 3-kinases(PI3K)/protein kinase B (Akt) mechanochemical transduction pathways and alter chondrocyte-induced ECM production. The effect of LIPUST on articular cartilage can be used as a new treatment for OA (Cheng K. et al., 2014). Mesenchymal stem cells (MSCs) with high expression of the α10 subunit have been proven to improve chondrogenic potential. Research showed that intra-articular injections of MSCs with high integrin α10 expression after joint damage may protect against posttraumatic OA (Delco et al., 2020). Another study showed that mechanical exposure at moderate intensity combined with diacerein treatment could modulate integrin-FAK-MAPK mechanotransduction in human osteoarthritis chondrocytes (Lohberger et al., 2019). In addition to the treatments mentioned above, we have summarized the results of recent experiments on the treatment of OA based on interference of integrin-mediated signaling pathways in the following table (Table 1).

**TABLE 1:** Recent experiments on treating OA by interfering with integrin-mediated signaling pathways.

**Interventions**	**Interfered integrin-mediated signaling pathway**	**Results**	**References**
Integrin-β-like 1 (Itgbl1)	Interact with integrins to down-regulate activity.	In patients with osteoarthritis (OA), the expression of Itgbl1 is greatly reduced. The ectopic expression of Itgbl1 can protect articular cartilage from the development of OA.	Song et al. (2018)
locus-1 (Del1)	Integrin α_V_β_3_-ERK/AKT signaling pathway.	DEL1 protected chondrocytes from apoptosis induced by various activators through integrin αVβ_3_-mediated signal pathways.	Wang et al. (2018)
Mechanical exposure and diacerein treatment	Integrin-FAK/STAT3-MAPKs signaling pathway.	In OA chondrocytes a significant reduction in the expression of Piezo1 was detected following treatment with diacerein, even in the presence of mechanical stimulation.	Lohberger et al. (2019)
Collagen type II (COL2A1)	Promote the interactions between integrin β1 and SMAD1.	COL2A1 can inhibit BMP-SMAD1-mediated chondrocyte hypertrophy.	Lian et al. (2019)
Cilengitide	Inhibit integrin αVβ3/αVβ5-FAK-MAPK signaling pathway.	Cilengitide can suppress inflammation in chondrocytes under excessive mechanical stress by interfering integrin-mediated signaling pathway	Hirose et al. (2020)
Cationic solid lipid nanoparticles loaded by integrin β1 plasmid DNA	Enforce the expression of integrin β1.	SLNs-pDNA treatment can reduce the apoptosis of rat chondrocytes and enhance tissue repair, which can be used as a potential non-drug in the treatment of OA.	Zhao Z. et al. (2020)
Exogenic TGF-β1 and WISP1 protein	Interact with Integrin α5 or Integrin αv.	TGF-β1 and WISP1 interact to induce CHs dedifferentiation, which was mainly mediated by integrin αV. However, Integrin αV showed a protective effect.	Zhang et al. (2020)
Vitronectin (VTN) fragment	Interact with α_V_β_6_ in human fibroblast-like synoviocytes.	VTN could prevent TGF-β1 activation by interacting with α_V_β_6_ in human FLSs and increase the level of α-SMA.	Ciregia et al. (2021)
Angiopoietin-like proteins (ANGPTLs)	Integrin α5β1- ERK/p38/JNK-NF-κB signaling pathway.	ANGPTL2 enhanced the gene expression of inflammatory mediators, while pretreatment with anti-LILRB2 antibody for 12 h reduced the inflammatory response.	Nishiyama et al. (2021)

## Conclusion

As transmembrane molecules on the cell surface, integrins play important roles in cartilage homeostasis, including cell survival, cell differentiation, matrix remodeling, and responses to mechanical stimulation. Integrins undergo many changes in OA, which may suggest that integrins are involved in the pathological procession of OA. Recent studies have proved that the important roles of integrins in OA cartilage, subchondral bone, and synovium. Integrin-mediated signaling pathways are key sources of the catabolic reactions critical for ECM destruction. Excessive mechanical loading can cause the destruction of the cartilage matrix, and abnormal mechanical signals from the ECM mediated by integrins work together to promote progressive destruction of the cartilage matrix in OA. Interactions between cytokines and integrins also contribute to the progression of OA. Changes in integrins also contribute to pathological changes in the subchondral bone and synovium. Integrin shows good application prospects for the treatment of OA. Interfering with integrin-mediated signaling pathways is a novel therapeutic approach to OA.

## Author Contributions

HJ, SJ, and RW conceptualized this review, decided on the content, wrote the manuscript, and prepared the figures. YL, JD, and YZ revised the manuscript. All authors approved the final version of the manuscript and agreed to be accountable for all aspects of the work.

## Conflict of Interest

The authors declare that the research was conducted in the absence of any commercial or financial relationships that could be construed as a potential conflict of interest.

## References

[B1] Adebayo O. O. Ko F. C. Wan P. T. Goldring S. R. Goldring M. B. Wright T. M. (2017). Role of subchondral bone properties and changes in development of load-induced osteoarthritis in mice. *Osteoarthr. Cartil.* 25 2108–2118. 10.1016/j.joca.2017.08.016 28919430 PMC5688000

[B2] Aigner T. Fundel K. Saas J. Gebhard P. M. Haag J. Weiss T. (2006). Large-scale gene expression profiling reveals major pathogenetic pathways of cartilage degeneration in osteoarthritis. *Arthritis Rheum.* 54 3533–3544. 10.1002/art.22174 17075858

[B3] Almonte-Becerril M. Costell M. Kouri J. B. (2014). Changes in the integrins expression are related with the osteoarthritis severity in an experimental animal model in rats. *J. Orthop. Res.* 32 1161–1166. 10.1002/jor.22649 24839051

[B4] Ansari A. A. Byrareddy S. N. (2016). The role of integrin expressing cells in modulating disease susceptibility and progression (January 2016). *Int. Trends Immun.* 4 11–27. 28770236 PMC5536173

[B5] Arner E. C. Tortorella M. D. (1995). Signal transduction through chondrocyte integrin receptors induces matrix metalloproteinase synthesis and synergizes with interleukin-1. *Arthritis Rheum.* 38 1304–1314. 10.1002/art.1780380919 7575726

[B6] Arokoski J. P. Jurvelin J. S. Väätäinen U. Helminen H. J. (2000). Normal and pathological adaptations of articular cartilage to joint loading. *Scand. J. Med. Sci. Sports* 10 186–198. 10.1034/j.1600-0838.2000.010004186.x 10898262

[B7] Attur M. G. Dave M. N. Clancy R. M. Patel I. R. Abramson S. B. Amin A. R. (2000). Functional genomic analysis in arthritis-affected cartilage: yin-yang regulation of inflammatory mediators by alpha 5 beta 1 and alpha V beta 3 integrins. *J. Immunol.* 164 2684–2691. 10.4049/jimmunol.164.5.2684 10679109

[B8] Au M. Liu Z. Rong L. Zheng Y. Wen C. (2020). Endothelin-1 induces chondrocyte senescence and cartilage damage via endothelin receptor type B in a post-traumatic osteoarthritis mouse model. *Osteoarthr. Cartil.* 28 1559–1571. 10.1016/j.joca.2020.08.006 32858189

[B9] Bernhard J. Ferguson J. Rieder B. Heimel P. Nau T. Tangl S. (2017). Tissue-engineered hypertrophic chondrocyte grafts enhanced long bone repair. *Biomaterials* 139 202–212. 10.1016/j.biomaterials.2017.05.045 28622604

[B10] Camper L. Hellman U. Lundgren-Akerlund E. (1998). Isolation, cloning, and sequence analysis of the integrin subunit alpha10, a beta1-associated collagen binding integrin expressed on chondrocytes. *J. Biol. Chem.* 273 20383–20389. 10.1074/jbc.273.32.20383 9685391

[B11] Carballo C. B. Nakagawa Y. Sekiya I. Rodeo S. A. (2017). Basic science of articular cartilage. *Clin. Sports Med.* 36 413–425. 28577703 10.1016/j.csm.2017.02.001

[B12] Castaño Betancourt M. C. Cailotto F. Kerkhof H. J. Cornelis F. M. Doherty S. A. Hart D. J. (2012). Genome-wide association and functional studies identify the DOT1L gene to be involved in cartilage thickness and hip osteoarthritis. *Proc. Natl. Acad. Sci. U. S. A.* 109 8218–8223. 10.1073/pnas.1119899109 22566624 PMC3361426

[B13] Chan P. M. B. Wen C. Yang W. C. Yan C. Chiu K. (2017). Is subchondral bone cyst formation in non-load-bearing region of osteoarthritic knee a vascular problem? *Med. Hypotheses* 109 80–83. 10.1016/j.mehy.2017.09.027 29150301

[B14] Chen D. Shen J. Zhao W. Wang T. Han L. Hamilton J. L. (2017). Osteoarthritis: toward a comprehensive understanding of pathological mechanism. *Bone Res.* 5:16044. 10.1038/boneres.2016.44 PMC5240031 28149655

[B15] Chen F. H. Thomas A. O. Hecht J. T. Goldring M. B. Lawler J. (2005). Cartilage oligomeric matrix protein/thrombospondin 5 supports chondrocyte attachment through interaction with integrins. *J. Biol. Chem.* 280 32655–32661. 10.1074/jbc.m504778200 16051604 PMC2896261

[B16] Cheng C. Gao S. Lei G. (2014). Association of osteopontin with osteoarthritis. *Rheumatol. Int.* 34 1627–1631. 10.1007/s00296-014-3036-9 24807695

[B17] Cheng J. Hu X. Dai L. Zhang X. Ren B. Shi W. (2016). Inhibition of transforming growth factor β-activated kinase 1 prevents inflammation-related cartilage degradation in osteoarthritis. *Sci. Rep.* 6:34497. 10.1038/srep34497 PMC5041103 27682596

[B18] Cheng K. Xia P. Lin Q. Shen S. Gao M. Ren S. (2014). Effects of low-intensity pulsed ultrasound on integrin-FAK-PI3K/Akt mechanochemical transduction in rabbit osteoarthritis chondrocytes. *Ultrasound Med. Biol.* 40 1609–1618. 10.1016/j.ultrasmedbio.2014.03.002 24742749

[B19] Ciregia F. Deroyer C. Cobraiville G. Plener Z. Malaise O. Gillet P. (2021). Modulation of α(V)β(6) integrin in osteoarthritis-related synovitis and the interaction with VTN((381-397 a.a.)) competing for TGF-β1 activation. *Exp. Mol. Med.* 53 210–222. 10.1038/s12276-021-00558-2 33526813 PMC8080589

[B20] Delco M. L. Goodale M. Talts J. F. Pownder S. L. Koff M. F. Miller A. D. (2020). Integrin α10β1-selected mesenchymal stem cells mitigate the progression of osteoarthritis in an equine talar impact model. *Am. J. Sports Med.* 48 612–623. 10.1177/0363546519899087 32004077

[B21] Dustin M. L. (2019). Integrins and their role in immune cell adhesion. *Cell* 177 499–501. 10.1016/j.cell.2019.03.038 30952447

[B22] Engleman V. W. Nickols G. A. Ross F. P. Horton M. A. Griggs D. W. Settle S. L. (1997). A peptidomimetic antagonist of the alpha(v)beta3 integrin inhibits bone resorption in vitro and prevents osteoporosis in vivo. *J. Clin. Invest.* 99 2284–2292. 10.1172/jci119404 9151803 PMC508061

[B23] Fang T. Zhou X. Jin M. Nie J. Li X. (2021). Molecular mechanisms of mechanical load-induced osteoarthritis. *Int. Orthop.* 45 1125–1136. 10.1007/s00264-021-04938-1 33459826

[B24] Finney A. C. Stokes K. Y. Pattillo C. B. Orr A. W. (2017). Integrin signaling in atherosclerosis. *Cell Mol. Life Sci.* 74 2263–2282. 10.1007/s00018-017-2490-4 28246700 PMC5427000

[B25] Forsyth C. B. Pulai J. Loeser R. F. (2002). Fibronectin fragments and blocking antibodies to alpha2beta1 and alpha5beta1 integrins stimulate mitogen-activated protein kinase signaling and increase collagenase 3 (matrix metalloproteinase 13) production by human articular chondrocytes. *Arthritis Rheum.* 46 2368–2376. 10.1002/art.10502 12355484

[B26] Gao Y. Liu S. Huang J. Guo W. Chen J. Zhang L. (2014). The ECM-cell interaction of cartilage extracellular matrix on chondrocytes. *Biomed. Res. Int.* 2014:648459. 10.1155/2014/648459 PMC4052144 24959581

[B27] Garciadiego-Cázares D. Aguirre-Sánchez H. I. Abarca-Buis R. F. Kouri J. B. Velasquillo C. Ibarra C. (2015). Regulation of α5 and αV integrin expression by GDF-5 and BMP-7 in chondrocyte differentiation and osteoarthritis. *PLoS One* 10:e0127166. 10.1371/journal.pone.0127166 26010756 PMC4443976

[B28] Gemba T. Valbracht J. Alsalameh S. Lotz M. (2002). Focal adhesion kinase and mitogen-activated protein kinases are involved in chondrocyte activation by the 29-kDa amino-terminal fibronectin fragment. *J. Biol. Chem.* 277 907–911. 10.1074/jbc.m109690200 11677248

[B29] Geoghegan I. P. Hoey D. A. McNamara L. M. (2019). Integrins in osteocyte biology and mechanotransduction. *Curr. Osteoporos. Rep.* 17 195–206. 10.1007/s11914-019-00520-2 31250372

[B30] Ginsberg M. H. (2014). Integrin activation. *BMB Rep.* 47 655–659. 25388208 10.5483/BMBRep.2014.47.12.241 PMC4345508

[B31] Goldring M. B. Goldring S. R. (2007). Osteoarthritis. *J. Cell. Physiol.* 213 626–634. 17786965 10.1002/jcp.21258

[B32] Goldring M. B. Goldring S. R. (2010). Articular cartilage and subchondral bone in the pathogenesis of osteoarthritis. *Ann. N. Y. Acad. Sci.* 1192 230–237. 10.1111/j.1749-6632.2009.05240.x 20392241

[B33] Grässel S. Muschter D. (2020). Recent advances in the treatment of osteoarthritis. *F1000Res* 9:F1000. 10.12688/f1000research.22115.1 PMC7199286 32419923

[B34] Guilak F. Nims R. J. Dicks A. Wu C. L. Meulenbelt I. (2018). Osteoarthritis as a disease of the cartilage pericellular matrix. *Matrix Biol.* 7 40–50. 10.1016/j.matbio.2018.05.008 29800616 PMC6146061

[B35] Hansen U. (2019). Analysis of collagen-binding integrin interactions with supramolecular aggregates of the extracellular matrix. *Methods Mol. Biol.* 1944 157–166. 10.1007/978-1-4939-9095-5_12 30840242

[B36] Häusler G. Helmreich M. Marlovits S. Egerbacher M. (2002). Integrins and extracellular matrix proteins in the human childhood and adolescent growth plate. *Calcif. Tissue Int.* 71 212–218. 10.1007/s00223-001-2083-x 12154393

[B37] Hirose N. Okamoto Y. Yanoshita M. Asakawa Y. Sumi C. Takano M. (2020). Protective effects of cilengitide on inflammation in chondrocytes under excessive mechanical stress. *Cell Biol. Int.* 44 966–974. 10.1002/cbin.11293 31876323

[B38] Homandberg G. A. (1999). Potential regulation of cartilage metabolism in osteoarthritis by fibronectin fragments. *Front. Biosci.* 4:D713-30. 10.2741/homandberg 10525477

[B39] Homandberg G. A. Meyers R. Williams J. M. (1993). Intraarticular injection of fibronectin fragments causes severe depletion of cartilage proteoglycans in vivo. *J. Rheumatol.* 20 1378–1382. 8230023

[B40] Horton M. A. Taylor M. L. Arnett T. R. Helfrich M. H. (1991). Arg-Gly-Asp (RGD) peptides and the anti-vitronectin receptor antibody 23C6 inhibit dentine resorption and cell spreading by osteoclasts. *Exp. Cell Res.* 195 368–375. 10.1016/0014-4827(91)90386-9 1712731

[B41] Huang Y. Z. Zhao L. Zhu Y. Tian S. J. Zhang W. Liu S. (2021). Interrupting TGF-β1/CCN2/integrin-α5β1 signaling alleviates high mechanical-stress caused chondrocyte fibrosis. *Eur. Rev. Med. Pharmacol. Sci.* 25 1233–1241. 33629293 10.26355/eurrev_202102_24827

[B42] Hügle T. Geurts J. (2017). What drives osteoarthritis?-synovial versus subchondral bone pathology. *Rheumatology* 56 1461–1471. 28003493 10.1093/rheumatology/kew389

[B43] Hui A. Y. McCarty W. J. Masuda K. Firestein G. S. Sah R. L. (2012). A systems biology approach to synovial joint lubrication in health, injury, and disease. *Wiley Interdiscip. Rev. Syst. Biol. Med.* 4 15–37. 10.1002/wsbm.157 21826801 PMC3593048

[B44] Humphries J. D. Chastney M. R. Askari J. A. Humphries M. J. (2019). Signal transduction via integrin adhesion complexes. *Curr. Opin. Cell Biol.* 56 14–21. 10.1016/j.ceb.2018.08.004 30195153

[B45] Jablonski C. L. Ferguson S. Pozzi A. Clark A. L. (2014). Integrin α1β1 participates in chondrocyte transduction of osmotic stress. *Biochem. Biophys. Res. Commun.* 445 184–190. 10.1016/j.bbrc.2014.01.157 24495803 PMC4022045

[B46] Jaiprakash A. Prasadam I. Feng J. Q. Liu Y. Crawford R. Xiao Y. (2012). Phenotypic characterization of osteoarthritic osteocytes from the sclerotic zones: a possible pathological role in subchondral bone sclerosis. *Int. J. Biol. Sci.* 8 406–417. 10.7150/ijbs.4221 22419886 PMC3303142

[B47] Jang K. W. Buckwalter J. A. Martin J. A. (2014). Inhibition of cell-matrix adhesions prevents cartilage chondrocyte death following impact injury. *J. Orthop. Res.* 32 448–454. 10.1002/jor.22523 24249698 PMC4034578

[B48] Jørgensen A. E. M. Kjær M. Heinemeier K. M. (2017). The effect of aging and mechanical loading on the metabolism of articular cartilage. *J. Rheumatol.* 44 410–417. 10.3899/jrheum.160226 28250141

[B49] Kadry Y. A. Calderwood D. A. (2020). Chapter 22: structural and signaling functions of integrins. *Biochim. Biophys. Acta Biomembr.* 1862:183206. 10.1016/j.bbamem.2020.183206 31991120 PMC7063833

[B50] Kong F. Li Z. Parks W. M. Dumbauld D. W. García A. J. Mould A. P. (2013). Cyclic mechanical reinforcement of integrin-ligand interactions. *Mol. Cell.* 49 1060–1068. 10.1016/j.molcel.2013.01.015 23416109 PMC3615084

[B51] Kozhemyakina E. Zhang M. Ionescu A. Ayturk U. M. Ono N. Kobayashi A. (2015). Identification of a Prg4-expressing articular cartilage progenitor cell population in mice. *Arthritis Rheumatol.* 67 1261–1273. 10.1002/art.39030 25603997 PMC4414823

[B52] Kurtis M. S. Schmidt T. A. Bugbee W. D. Loeser R. F. Sah R. L. (2003). Integrin-mediated adhesion of human articular chondrocytes to cartilage. *Arthritis Rheum.* 48 110–118. 10.1002/art.10704 12528111

[B53] Lahiji K. Polotsky A. Hungerford D. S. Frondoza C. G. (2004). Cyclic strain stimulates proliferative capacity, alpha2 and alpha5 integrin, gene marker expression by human articular chondrocytes propagated on flexible silicone membranes. *Vitro Cell Dev. Biol. Anim.* 40 138–142. 10.1290/1543-706x(2004)40<138:csspca>2.0.co;2 15479117

[B54] Lapadula G. Iannone F. Zuccaro C. Grattagliano V. Covelli M. Patella V. (1997). Integrin expression on chondrocytes: correlations with the degree of cartilage damage in human osteoarthritis. *Clin. Exp. Rheumatol.* 15 247–254. 9177918

[B55] Lawrence T. (2009). The nuclear factor NF-kappaB pathway in inflammation. *Cold Spring Harb. Perspect. Biol.* 1:a001651. 10.1101/cshperspect.a001651 PMC2882124 20457564

[B56] Lee A. S. Ellman M. B. Yan D. Kroin J. S. Cole B. J. van Wijnen A. J. (2013). A current review of molecular mechanisms regarding osteoarthritis and pain. *Gene* 527 440–447. 10.1016/j.gene.2013.05.069 23830938 PMC3745800

[B57] Lee H. S. Millward-Sadler S. J. Wright M. O. Nuki G. Salter D. M. (2000). Integrin and mechanosensitive ion channel-dependent tyrosine phosphorylation of focal adhesion proteins and beta-catenin in human articular chondrocytes after mechanical stimulation. *J. Bone Miner. Res.* 15 1501–1509. 10.1359/jbmr.2000.15.8.1501 10934648

[B58] Legate K. R. Wickström S. A. Fässler R. (2009). Genetic and cell biological analysis of integrin outside-in signaling. *Genes Dev.* 23 397–418. 10.1101/gad.1758709 19240129

[B59] Leitinger B. Kwan A. P. (2006). The discoidin domain receptor DDR2 is a receptor for type X collagen. *Matrix Biol.* 25 355–364. 10.1016/j.matbio.2006.05.006 16806867

[B60] Li G. Yin J. Gao J. Cheng T. S. Pavlos N. J. Zhang C. (2013). Subchondral bone in osteoarthritis: insight into risk factors and microstructural changes. *Arthritis Res. Ther.* 15:223. 10.1186/ar4405 24321104 PMC4061721

[B61] Li L. Newton P. T. Bouderlique T. Sejnohova M. Zikmund T. Kozhemyakina E. (2017). Superficial cells are self-renewing chondrocyte progenitors, which form the articular cartilage in juvenile mice. *FASEB J.* 31 1067–1084. 10.1096/fj.201600918r 27965322 PMC5295727

[B62] Lian C. Wang X. Qiu X. Wu Z. Gao B. Liu L. (2019). Collagen type II suppresses articular chondrocyte hypertrophy and osteoarthritis progression by promoting integrin β1-SMAD1 interaction. *Bone Res.* 7:8. 10.1038/s41413-019-0046-y PMC6403405 30854241

[B63] Liang J. Xu L. Zhou F. Liu A. M. Ge H. X. Chen Y. Y. (2018). MALAT1/miR-127-5p regulates osteopontin (OPN)-mediated proliferation of human chondrocytes through PI3K/Akt pathway. *J. Cell. Biochem.* 119 431–439. 10.1002/jcb.26200 28590075

[B64] Liang W. Li Z. Wang Z. Zhou J. Song H. Xu S. (2017). Periodic mechanical stress INDUCES chondrocyte proliferation and matrix synthesis via CaMKII-mediated Pyk2 signaling. *Cell Physiol. Biochem.* 42 383–396. 10.1159/000477483 28558386

[B65] Liu Z. Au M. Wang X. Chan P. B. Lai P. Sun L. (2019). Photoacoustic imaging of synovial tissue hypoxia in experimental post-traumatic osteoarthritis. *Prog. Biophys. Mol. Biol.* 148 12–20. 10.1016/j.pbiomolbio.2018.03.009 29601835

[B66] Loeser R. F. (1993). Integrin-mediated attachment of articular chondrocytes to extracellular matrix proteins. *Arthritis Rheum.* 36 1103–1110. 10.1002/art.1780360811 8343186

[B67] Loeser R. F. (1997). Growth factor regulation of chondrocyte integrins. differential effects of insulin-like growth factor 1 and transforming growth factor beta on alpha 1 beta 1 integrin expression and chondrocyte adhesion to type VI collagen. *Arthritis Rheum.* 40 270–276. 10.1002/art.1780400211 9041938

[B68] Loeser R. F. (2000). Chondrocyte integrin expression and function. *Biorheology* 37 109–116. 10912183

[B69] Loeser R. F. (2002). Integrins and cell signaling in chondrocytes. *Biorheology* 39 119–124. 12082274

[B70] Loeser R. F. (2009). Aging and osteoarthritis: the role of chondrocyte senescence and aging changes in the cartilage matrix. *Osteoarthr. Cartil.* 17 971–979. 10.1016/j.joca.2009.03.002 19303469 PMC2713363

[B71] Loeser R. F. (2014). Integrins and chondrocyte-matrix interactions in articular cartilage. *Matrix Biol.* 39 11–16. 10.1016/j.matbio.2014.08.007 25169886 PMC4699681

[B72] Loeser R. F. Carlson C. S. McGee M. P. (1995). Expression of beta 1 integrins by cultured articular chondrocytes and in osteoarthritic cartilage. *Exp. Cell Res.* 217 248–257. 10.1006/excr.1995.1084 7535235

[B73] Loeser R. F. Erickson E. A. Long D. L. (2008). Mitogen-activated protein kinases as therapeutic targets in osteoarthritis. *Curr. Opin. Rheumatol.* 20 581–586. 10.1097/bor.0b013e3283090463 18698181 PMC2892710

[B74] Loeser R. F. Sadiev S. Tan L. Goldring M. B. (2000). Integrin expression by primary and immortalized human chondrocytes: evidence of a differential role for alpha1beta1 and alpha2beta1 integrins in mediating chondrocyte adhesion to types II and VI collagen. *Osteoarthr. Cartil.* 8 96–105. 10.1053/joca.1999.0277 10772239

[B75] Lohberger B. Kaltenegger H. Weigl L. Mann A. Kullich W. Stuendl N. (2019). Mechanical exposure and diacerein treatment modulates integrin-FAK-MAPKs mechanotransduction in human osteoarthritis chondrocytes. *Cell. Signal.* 56 23–30. 10.1016/j.cellsig.2018.12.010 30583016

[B76] Lowin T. Straub R. H. (2011). Integrins and their ligands in rheumatoid arthritis. *Arthritis Res. Ther.* 13:244. 10.1186/ar3464 22077951 PMC3308078

[B77] Lowin T. Straub R. H. Neumann E. Bosserhoff A. Vogel C. Moissl C. (2009). Glucocorticoids increase alpha5 integrin expression and adhesion of synovial fibroblasts but inhibit ERK signaling, migration, and cartilage invasion. *Arthritis Rheum.* 60 3623–3632. 10.1002/art.24985 19950288

[B78] Lucchinetti E. Bhargava M. M. Torzilli P. A. (2004). The effect of mechanical load on integrin subunits alpha5 and beta1 in chondrocytes from mature and immature cartilage explants. *Cell Tissue Res.* 315 385–391. 10.1007/s00441-003-0836-8 14673641

[B79] Margadant C. Sonnenberg A. (2010). Integrin-TGF-beta crosstalk in fibrosis, cancer and wound healing. *EMBO Rep.* 11 97–105. 10.1038/embor.2009.276 20075988 PMC2828749

[B80] Marie P. J. Haÿ E. Saidak Z. (2014). Integrin and cadherin signaling in bone: role and potential therapeutic targets. *Trends Endocrinol. Metab.* 25 567–575. 10.1016/j.tem.2014.06.009 25034128

[B81] Marini J. C. Forlino A. Bächinger H. P. Bishop N. J. Byers P. H. Paepe A. (2017). Osteogenesis imperfecta. *Nat. Rev. Dis. Primers* 3:17052. 10.1038/nrdp.2017.52 28820180

[B82] Michael M. Parsons M. (2020). New perspectives on integrin-dependent adhesions. *Curr. Opin. Cell Biol.* 63 31–37. 10.1016/j.ceb.2019.12.008 31945690 PMC7262580

[B83] Millward-Sadler S. J. Salter D. M. (2004). Integrin-dependent signal cascades in chondrocyte mechanotransduction. *Ann. Biomed. Eng.* 32 435–446. 10.1023/b:abme.0000017538.72511.48 15095818

[B84] Monfort J. Garcia-Giralt N. López-Armada M. J. Monllau J. C. Bonilla A. Benito P. (2006). Decreased metalloproteinase production as a response to mechanical pressure in human cartilage: a mechanism for homeostatic regulation. *Arthritis Res. Ther.* 8:R149. 10.1186/ar2042 PMC1779454 16972994

[B85] Morshed A. Abbas A. B. Hu J. Xu H. (2019). Shedding new light on the role of ανβ3 and α5β1 integrins in rheumatoid arthritis. *Molecules* 24:1537. 10.3390/molecules24081537 31003546 PMC6515208

[B86] Nishiyama S. Hirose N. Yanoshita M. Takano M. Kubo N. Yamauchi Y. (2021). ANGPTL2 induces synovial inflammation via LILRB2. *Inflammation* 44 1108–1118. 10.1007/s10753-020-01406-7 33538932

[B87] Orazizadeh M. Salter D. M. (2008). CD147 (extracellular matrix metalloproteinase inducer-emmprin) expression by human articular chondrocytes. *Iran. Biomed. J.* 12 153–158. 18762818

[B88] Orazizadeh M. Lee H. S. Groenendijk B. Sadler S. J. Wright M. O. Lindberg F. P. (2008). CD47 associates with alpha 5 integrin and regulates responses of human articular chondrocytes to mechanical stimulation in an in vitro model. *Arthritis Res. Ther.* 10:R4. 10.1186/ar2350 PMC2374443 18186923

[B89] Ostergaard K. Salter D. M. Petersen J. Bendtzen K. Hvolris J. Andersen C. B. (1998). Expression of alpha and beta subunits of the integrin superfamily in articular cartilage from macroscopically normal and osteoarthritic human femoral heads. *Ann. Rheum. Dis.* 57 303–308. 10.1136/ard.57.5.303 9741315 PMC1752603

[B90] Palazzo C. Nguyen C. Lefevre-Colau M. M. Rannou F. Poiraudeau S. (2016). Risk factors and burden of osteoarthritis. *Ann. Phys. Rehabil. Med.* 59 134–138. 10.1016/j.rehab.2016.01.006 26904959

[B91] Perera P. M. Wypasek E. Madhavan S. Rath-Deschner B. Liu J. Nam J. (2010). Mechanical signals control SOX-9, VEGF, and c-Myc expression and cell proliferation during inflammation via integrin-linked kinase, B-Raf, and ERK1/2-dependent signaling in articular chondrocytes. *Arthritis Res. Ther.* 12:R106. 10.1186/ar3039 PMC2911896 20509944

[B92] Pérez-García S. Carrión M. Gutiérrez-Cañas I. Villanueva-Romero R. Castro D. Martínez C. (2019). Profile of matrix-remodeling proteinases in osteoarthritis: impact of fibronectin. *Cells* 9:40. 10.3390/cells9010040 31877874 PMC7017325

[B93] Pirilä L. Aho H. Roivainen A. Konttinen Y. T. Pelliniemi L. J. Heino J. (2001). Identification of alpha6beta1 integrin positive cells in synovial lining layer as type B synoviocytes. *J. Rheumatol.* 28 478–484. 11296945

[B94] Poniatowski ŁA. Wojdasiewicz P. Krawczyk M. Szukiewicz D. Gasik R. Kubaszewski Ł (2017). Analysis of the role of CX3CL1 (Fractalkine) and its receptor CX3CR1 in traumatic brain and spinal cord injury: insight into recent advances in actions of neurochemokine agents. *Mol. Neurobiol.* 54 2167–2188. 10.1007/s12035-016-9787-4 26927660 PMC5355526

[B95] Prasadam I. Farnaghi S. Feng J. Q. Gu W. Perry S. Crawford R. (2013). Impact of extracellular matrix derived from osteoarthritis subchondral bone osteoblasts on osteocytes: role of integrinβ1 and focal adhesion kinase signaling cues. *Arthritis Res. Ther.* 15:R150. 10.1186/ar4333 PMC3978998 24289792

[B96] Prein C. Beier F. (2019). ECM signaling in cartilage development and endochondral ossification. *Curr. Top. Dev. Biol.* 133 25–47. 10.1016/bs.ctdb.2018.11.003 30902255

[B97] Rahmati M. Nalesso G. Mobasheri A. Mozafari M. (2017). Aging and osteoarthritis: central role of the extracellular matrix. *Ageing Res. Rev.* 40 20–30. 10.1016/j.arr.2017.07.004 28774716

[B98] Ren K. Tang J. Jiang X. Sun H. Nong L. Shen N. (2018). Periodic mechanical stress stimulates GIT1-dependent mitogenic signals in rat chondrocytes through ERK1/2 activity. *Cell Physiol. Biochem.* 50 1015–1028. 10.1159/000494513 30355914

[B99] Rinaldi N. Weis D. Brado B. Schwarz-Eywill M. Lukoschek M. Pezzutto A. (1997). Differential expression and functional behaviour of the alpha v and beta 3 integrin subunits in cytokine stimulated fibroblast-like cells derived from synovial tissue of rheumatoid arthritis and osteoarthritis in vitro. *Ann. Rheum. Dis.* 56 729–736. 10.1136/ard.56.12.729 9496152 PMC1752301

[B100] Roca-Cusachs P. Iskratsch T. Sheetz M. P. (2012). Finding the weakest link: exploring integrin-mediated mechanical molecular pathways. *J. Cell Sci.* 125(Pt 13) 3025–3038. 22797926 10.1242/jcs.095794 PMC6518164

[B101] Romero S. Le Clainche C. Gautreau A. M. (2020). Actin polymerization downstream of integrins: signaling pathways and mechanotransduction. *Biochem. J.* 477 1–21. 10.1042/bcj20170719 31913455

[B102] Sanchez C. Pesesse L. Gabay O. Delcour J. P. Msika P. Baudouin C. (2012). Regulation of subchondral bone osteoblast metabolism by cyclic compression. *Arthritis Rheum.* 64 1193–1203. 10.1002/art.33445 22034083

[B103] Scanzello C. R. Goldring S. R. (2012). The role of synovitis in osteoarthritis pathogenesis. *Bone* 51 249–257. 10.1016/j.bone.2012.02.012 22387238 PMC3372675

[B104] Schaffner P. Dard M. M. (2003). Structure and function of RGD peptides involved in bone biology. *Cell Mol. Life Sci.* 60 119–132. 10.1007/s000180300008 12613662 PMC11138839

[B105] Shan W. Cheng C. Huang W. Ding Z. Luo S. Cui G. (2019). Angiopoietin-like 2 upregulation promotes human chondrocyte injury via NF-κB and p38/MAPK signaling pathway. *J. Bone Miner. Metab.* 37 976–986. 10.1007/s00774-019-01016-w 31214838

[B106] Sharifi N. Gharravi A. M. (2019). Shear bioreactors stimulating chondrocyte regeneration, a systematic review. *Inflamm. Regen.* 39:16. 10.1186/s41232-019-0105-1 PMC6686520 31410225

[B107] Shattil S. J. Kim C. Ginsberg M. H. (2010). The final steps of integrin activation: the end game. *Nat. Rev. Mol. Cell Biol.* 11 288–300. 10.1038/nrm2871 20308986 PMC3929966

[B108] Sheng Z. Cao X. Peng S. Wang C. Li Q. Wang Y. (2008). Ofloxacin induces apoptosis in microencapsulated juvenile rabbit chondrocytes by caspase-8-dependent mitochondrial pathway. *Toxicol. Appl. Pharmacol.* 226 119–127. 10.1016/j.taap.2007.08.025 17961619

[B109] Silver F. H. Bradica G. Tria A. (2001). Relationship among biomechanical, biochemical, and cellular changes associated with osteoarthritis. *Crit. Rev. Biomed. Eng.* 29 373–391. 10.1615/critrevbiomedeng.v29.i4.10 11822479

[B110] Smith R. L. Carter D. R. Schurman D. J. (2004). Pressure and shear differentially alter human articular chondrocyte metabolism: a review. *Clin. Orthop. Relat. Res.* 427(Suppl.) S89–S95. 15480081

[B111] Sofat N. (2009). Analysing the role of endogenous matrix molecules in the development of osteoarthritis. *Int. J. Exp. Pathol.* 90 463–479. 10.1111/j.1365-2613.2009.00676.x 19765101 PMC2768145

[B112] Song E. K. Jeon J. Jang D. G. Kim H. E. Sim H. J. Kwon K. Y. (2018). ITGBL1 modulates integrin activity to promote cartilage formation and protect against arthritis. *Sci. Transl. Med.* 10:eaam7486. 10.1126/scitranslmed.aam7486 30305454

[B113] Su C. M. Chiang Y. C. Huang C. Y. Hsu C. J. Fong Y. C. Tang C. H. (2015). Osteopontin promotes oncostatin M production in human osteoblasts: implication of rheumatoid arthritis therapy. *J. Immunol.* 195 3355–3364. 10.4049/jimmunol.1403191 26304992

[B114] Sun Z. Costell M. Fässler R. (2019). Integrin activation by talin, kindlin and mechanical forces. *Nat. Cell Biol.* 21 25–31. 10.1038/s41556-018-0234-9 30602766

[B115] Takada Y. Ye X. Simon S. (2007). The integrins. *Genome Biol.* 8:215. 10.1186/gb-2007-8-5-215 PMC1929136 17543136

[B116] Takano M. Hirose N. Sumi C. Yanoshita M. Nishiyama S. Onishi A. (2019). ANGPTL2 promotes inflammation via integrin α5β1 in chondrocytes. *Cartilage* 10.1177/1947603519878242 Online ahead of print. 31581797 PMC8804837

[B117] Ulrich-Vinther M. Maloney M. D. Schwarz E. M. Rosier R. O’Keefe R. J. (2003). Articular cartilage biology. *J. Am. Acad. Orthop. Surg.* 11 421–430. 14686827 10.5435/00124635-200311000-00006

[B118] Vazquez K. J. Andreae J. T. Henak C. R. (2019). Cartilage-on-cartilage cyclic loading induces mechanical and structural damage. *J. Mech. Behav. Biomed. Mater.* 98 262–267. 10.1016/j.jmbbm.2019.06.023 31280053 PMC6698399

[B119] Wang Q. Onuma K. Liu C. Wong H. Bloom M. S. Elliott E. E. (2019). Dysregulated integrin αVβ3 and CD47 signaling promotes joint inflammation, cartilage breakdown, and progression of osteoarthritis. *JCI Insight* 4:e128616. 10.1172/jci.insight.128616 PMC6795293 31534047

[B120] Wang Z. Boyko T. Tran M. C. LaRussa M. Bhatia N. Rashidi V. (2018). DEL1 protects against chondrocyte apoptosis through integrin binding. *J. Surg. Res.* 231 1–9. 10.1016/j.jss.2018.04.066 30278915

[B121] Weber A. Chan P. M. B. Wen C. (2019). Do immune cells lead the way in subchondral bone disturbance in osteoarthritis? *Prog. Biophys. Mol. Biol.* 148 21–31. 10.1016/j.pbiomolbio.2017.12.004 29277342

[B122] Wen C. Y. Wu C. B. Tang B. Wang T. Yan C. H. Lu W. W. (2012). Collagen fibril stiffening in osteoarthritic cartilage of human beings revealed by atomic force microscopy. *Osteoarthr. Cartil.* 20 916–922. 10.1016/j.joca.2012.04.018 22548795

[B123] Werb Z. Tremble P. M. Behrendtsen O. Crowley E. Damsky C. H. (1989). Signal transduction through the fibronectin receptor induces collagenase and stromelysin gene expression. *J. Cell Biol.* 109 877–889. 10.1083/jcb.109.2.877 2547805 PMC2115739

[B124] Woltersdorf C. Bonk M. Leitinger B. Huhtala M. Käpylä J. Heino J. (2017). The binding capacity of α1β 1-, α2β1- and α10β1-integrins depends on non-collagenous surface macromolecules rather than the collagens in cartilage fibrils. *Matrix Biol.* 63 91–105. 10.1016/j.matbio.2017.02.001 28192200

[B125] Wright M. O. Nishida K. Bavington C. Godolphin J. L. Dunne E. Walmsley S. (1997). Hyperpolarisation of cultured human chondrocytes following cyclical pressure-induced strain: evidence of a role for alpha 5 beta 1 integrin as a chondrocyte mechanoreceptor. *J. Orthop. Res.* 15 742–747. 10.1002/jor.1100150517 9420605

[B126] Zaidel-Bar R. Cohen M. Addadi L. Geiger B. (2004). Hierarchical assembly of cell-matrix adhesion complexes. *Biochem. Soc. Trans.* 32 416–420. 10.1042/bst0320416 15157150

[B127] Zemmyo M. Meharra E. J. Kühn K. Creighton-Achermann L. Lotz M. (2003). Accelerated, aging-dependent development of osteoarthritis in alpha1 integrin-deficient mice. *Arthritis Rheum.* 48 2873–2880. 10.1002/art.11246 14558093

[B128] Zhang C. Chiu K. Y. Chan B. P. M. Li T. Wen C. Xu A. (2018). Knocking out or pharmaceutical inhibition of fatty acid binding protein 4 (FABP4) alleviates osteoarthritis induced by high-fat diet in mice. *Osteoarthr. Cartil.* 26 824–833. 10.1016/j.joca.2018.03.002 29549054

[B129] Zhang M. Meng Q. C. Yang X. F. Mu W. D. (2020). TGF-β1/WISP1/Integrin-α interaction mediates human chondrocytes dedifferentiation. *Eur. Rev. Med. Pharmacol. Sci.* 24 8675–8684. 32964955 10.26355/eurrev_202009_22804

[B130] Zhao Y. Chen H. Wang L. Guo Z. Liu S. Luo S. (2020). Cationic solid lipid nanoparticles loaded by integrin β1 plasmid DNA attenuates IL-1β-induced apoptosis of chondrocyte. *Aging* 12 22527–22537. 33289706 10.18632/aging.103656 PMC7746374

[B131] Zhao Z. Li Y. Wang M. Zhao S. Zhao Z. Fang J. (2020). Mechanotransduction pathways in the regulation of cartilage chondrocyte homoeostasis. *J. Cell Mol. Med.* 24 5408–5419. 10.1111/jcmm.15204 32237113 PMC7214151

[B132] Zhen G. Guo Q. Li Y. Wu C. Zhu S. Wang R. (2021). Mechanical stress determines the configuration of TGFβ activation in articular cartilage. *Nat. Commun.* 12:1706. 10.1038/s41467-021-21948-0 PMC7969741 33731712

